# The transmission of SARS-CoV-2 is likely comodulated by temperature and by relative humidity

**DOI:** 10.1371/journal.pone.0255212

**Published:** 2021-07-29

**Authors:** Kevin S. Raines, Sebastian Doniach, Gyan Bhanot

**Affiliations:** 1 Cra 13 9aN 58, Armenia, Quindío, Colombia; 2 Applied Physics, Stanford University, Stanford, CA, United States of America; 3 Molecular Biology and Biochemistry, Rutgers University, Piscataway, NJ, United States of America; 4 Physics and Astronomy, Rutgers University, Piscataway, NJ, United States of America; 5 Rutgers Cancer Institute of New Jersey, New Brunswick, NJ, United States of America; 6 School of Medicine, University of California San Diego, La Jolla, CA, United States of America; Nanyang Technological University, SINGAPORE

## Abstract

Inferring the impact of climate upon the transmission of SARS-CoV-2 has been confounded by variability in testing, unknown disease introduction rates, and changing weather. Here we present a data model that accounts for dynamic testing rates and variations in disease introduction rates. We apply this model to data from Colombia, whose varied and seasonless climate, central port of entry, and swift, centralized response to the COVID-19 pandemic present an opportune environment for assessing the impact of climate factors on the spread of COVID-19. We observe strong attenuation of transmission in climates with sustained daily temperatures above 30 degrees Celsius and simultaneous mean relative humidity below 78%, with outbreaks occurring at high humidity even where the temperature is high. We hypothesize that temperature and relative humidity comodulate the infectivity of SARS-CoV-2 within respiratory droplets.

## Introduction

Coronaviruses are a class of large, enveloped, single-strand RNA viruses that are widespread in animals and provoke respiratory illnesses in humans [1]. The novel coronavirus SARS-CoV-2 was identified in January 2020 as the likely causative agent of a cluster of pneumonia cases appearing in Wuhan, China throughout December 2019, making it the seventh known coronavirus to cause pathology in humans [2]. SARS-CoV-2 is associated with a respiratory illness, COVID-19, that ranges in severity from an asymptomatic infection [3], to common-cold like symptoms, to viral pneumonia, acute respiratory distress syndrome, and death [4]. While the mortality in SARS-CoV-2 appears to be lower than in SARS-CoV [4], this new virus has more effective transmission characteristics [5], including an asymptomatic infective phase [6].

From early January to March 2020, SARS-CoV-2 quickly spread around the world, causing a global pandemic [7]. While global data will certainly play a role in elucidating the epidemiology of COVID-19, the data is confounded by several factors, such as country specific responses to the pandemic, varying degrees of compliance [7, 8], variations in testing availability and policies [9], varying mobility and travel by the population [10], seasonal climate in the two hemispheres, etc.

However, the analysis of the data is greatly simplified when only a single country is studied. Colombia is uniquely suited for the study of weather factors on the transmission of SARS-CoV-2 for the following five reasons:

1. *Climate variation*. Colombia has five geographically distinct regions: The Pacific coastal region, the Caribbean coastal region, the Andean mountain region, *los llanos* (grassland plains), and the Amazon Rainforest region. The unique and seasonless climate in each of these regions alleviates the confounding role of seasons on data from the hemispheres [11].
2. *Central port of entry*. The El Dorado International airport in Bogotá is by far the largest transportation hub for international travel, with nearly seven times the number of international travelers as the next largest airport in Colombia.
3. *Conditions favorable to rapid spread*. Colombian cities have high urban population densities (Table 1), and public transportation is widely used in Colombia, with only one fifth the number of registered cars per citizen compared to the USA. For example, in Barranquilla, a coastal city in the Caribbean with hot temperatures, the average weekday commute time on public transportation is 77 minutes. The heavy use of crowded public transportation means that the measured lack of spread of the virus in this city is not the result of social distancing among the populace.
4. *Lack of air-conditioning*. Indoor air-conditioning is rare in Colombia [12]. The citizens live in the ambient conditions of temperature and humidity of their environment. This eliminates individual specific variation in temperature and humidity as a potential confounding factor.
5. *Swift and coordinated national response*. The first COVID-19 case was confirmed in Colombia on March 6, 2020. Nineteen days later, the government implemented a national quarantine, with tight cooperation at the local level and testing orchestrated centrally through Bogotá [13]. This strong, centralized and swift action at the national level greatly simplifies the analysis because the data cleanly separates into pre-quarantine and post-quarantine periods.

**Table 1:** Geographic and population data for the five largest cities in Colombia.

	population	pop. density	max. daily temp	daily humidity
Bogotá	7,387,400	19,475	19.2 (1.2)	83.9 (8.0)
Medellín	2,382,399	20,681	25.2 (1.8)	83.5 (13.2)
Cali	2,172,527	16,247	26.0 (2.4)	81.8 (9.9)
Barranquilla	1,205,284	14,455	32.0 (0.7)	69.3 (10.3)
Cartagena	876,885	17,053	29.6 (0.6)	75.8 (7.2)

The temperature shown is the mean maximum daily temperature over the period of the study. In parentheses, the variation from the mean (the standard deviation) is shown. Our analysis found that the mean *maximum* daily temperature is an important proxy for the heat transmission barrier. The small variations demonstrate the constant weather patterns within each city studied as pertains to our model of SARS-CoV-2 transmission. The humidity is the mean daily relative humidity over the period of the study (standard deviation in parentheses). The population density is the urban population density.

**Fig 1:**
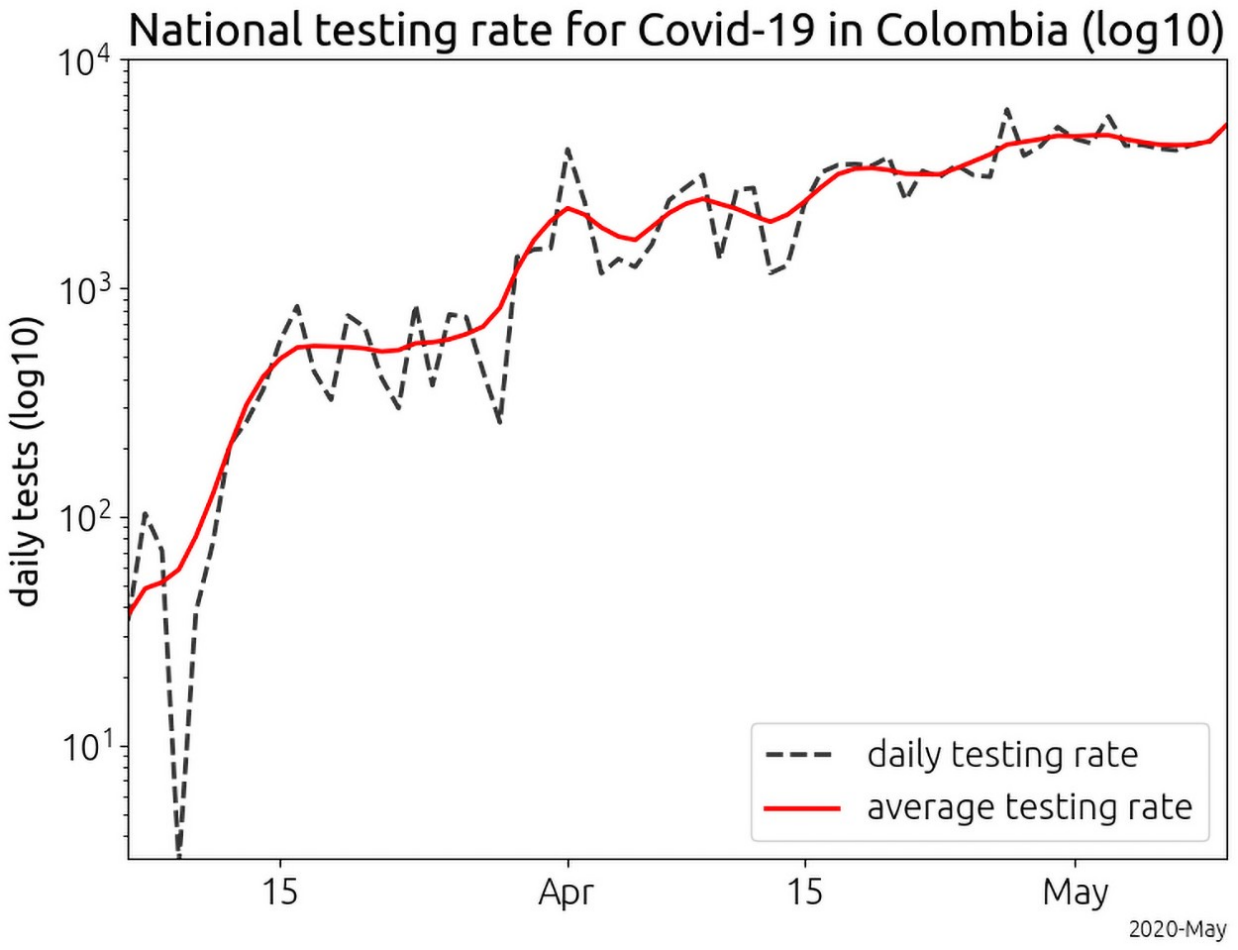
Average testing rates in Colombia, March through early May. The number of daily tests for SARS-CoV-2 increased by two orders of magnitude over two months. The testing rates shown are a running average computed with a centered, seven day window. We have identified dynamic testing rates as a key confounding factor in the analysis of COVID-19 case data.

The *transmission rate*, or the propensity of the disease to infect an exposed potential host, must be distinguished from both total disease prevalence and the number of confirmed cases in a given population. As we show in the supplementary text, the the number confirmed cases is confounded by the detection rate and the drip rate, or the rate at which infectious travelers arrive in a city. *Dynamic* detection rates caused by increasing disease awareness and testing introduce an additive factor into the exponent of the apparent transmission rate (S1 File). Over the course of our study, the daily testing rate in Colombia increased by over two orders of magnitude (Fig 1). Our data model accounts for variations in the drip rate and dynamic detection rates by considering confirmed case dynamics instead of total confirmed case numbers, since totals are confounded by public perception, varying testing rates, varying drip rates and stochastic fluctuations (S1 File).

Models of seasonal, viral respiratory illness demonstrate airborne respiratory virus transmissions exhibit a strong spatial decline (i.e. Gaussian) in the transmission rate with the distance between the recipient and the diseased host, and strongly depend upon local environmental factors such as air flow, temperature and humidity [14–18]. The strong dependence of the probability of transmission on host-recipient distance underscores the need to separate the data into pre-quarantine and post-quarantine periods, because the degree of social distancing changed dramatically post-quarantine. After quarantine, transmission rates decline in proportion to the strictness of the quarantine measures and the degree of compliance with them.

The transmission of a respiratory virus can be divided into four basic steps [19]: (1) a non-infected host interacts with the environment of an infected individual (2) the infected individual transmits *intact* virions to the non-infected host and (3) the virus infects the host (4) the virus replicates sufficiently for the host to become infectious.

In the first step, interaction, it is not necessary that the recipient and donor are in the infective environment simultaneously because, in a suitable environment, virus-droplets can remain suspended in the air for hours. Eventually, viruses inactivate due to the accumulation of environmental damage. This inactivation timescale governs the second step, which is viral transmission. The probability of infection, the third step, depends on the number of intact virions that deposit on the uninfected host and the susceptibility of the host. Susceptibility to infection depends on many factors, such as age, medical history, obesity and the internal humidity of the host, since a dry respiratory tract is more infection prone [20]. In the fourth step, replication, both demographic and environmental factors play a role. For example, temperature correlates with viral titer (hotter temperatures producing less titer [15]).

Consequently, the probabilities of these four steps are not independent. Environmental factors influence the frequency and conditions under which people associate, they regulate virus decomposition rates, modulate host susceptibility and severity of infection within the host. Droplet settling times, droplet evaporation times, and viral stability times, all depend on environmental factors such as humidity, temperature, wind etc.

Since all of these factors influence the transmission rate, they either must be included in the modeling, which introduces additional parameters and more complexity, or one can restrict the analysis to situations where the transmission occurs in environmental, social and economic steady-state conditions. The pre-quarantine Colombian data meets the steady-state conditions because of the lack of seasonality, the widespread daily use of transportation in densely packed buses and subways, the high population density in cities (Table 1) and the suddenness and totality of the imposed quarantine.

While several papers have been published on the topic of climate and COVID-19 transmission, ours is unique for the following reasons:

1. *First to observe a heat transmission barrier and a humidity dependence in the transmission of COVID-19*: Our paper, first posted online in May 2020, was one of the first to predict the climate dependence of COVID-19 from empirical observations.
2. *First to model testing*: One of the novel aspects of the COVID-19 pandemic was the steady increase in testing capacity as the pandemic progressed. To our knowledge, the impact of an ongoing increase in testing capacity had not been considered in the literature prior to our research.
3. *First to model disease spread by ongoing international travel*. Another novel aspect of the COVID-19 pandemic was the ongoing spread of the disease due to international travel after the pandemic had begun. Prior to COVID-19, the majority of models assumed a rapid quarantine or rapid ban on international travel, resulting in equations that modeled an initial seeding of the disease followed by spread. However, due to the slow international response with COVID-19, many countries had an ongoing influx of infectious travelers for months after their initial patient. To our knowledge, our paper was the first to model this phenomenon and the first to predict that the daily rate at which new infectious travelers arrive leads to a multiplicative factor in the total number of cases.
4. *First to control for mobility*. The majority of papers on the correlation between climate and COVID-19 transmission do not control for variations in population mobility among the various regions. Indeed, some research [21] points out that human mobility is known to affect temperature dependence of transmission because more people travel in warmer weather [22–25], leading to higher contact rates, and thereby offsetting the reduction in transmission due to higher temperatures that we identified in our paper. In our analysis, we control for mobility by only considering a single country (Colombia) with multiple climatic regions, each with distinct temperature and humidity patterns, and minimal movement of peoples between regions.
5. *First to propose a mechanism for the heat transmission barrier*: By considering the timescale of droplet evaporation and the previous literature on biophysical mechanisms of respiratory virus transmission, we were able to identify a potential mechanism for the observed heat transmission barrier. This hypothesis can be tested in laboratory, which is the next logical step for the non-mathematical part of the paper.

## Materials and methods

### The drip model

A significant fraction of infective hosts of SARS-CoV-2 are asymptomatic [5, 6]. In addition, SARS-CoV-2 has a long incubation period of up to ∼ 15 days [26]. During this time, the disease host is infectious but asymptomatic. In Colombia, international travel was banned three days after the imposition of national quarantine [13]. Consequently, we approximate that the rate at which infectious travelers arrived in each city was roughly constant over the pre-quarantine period.

We model the daily arrival of SARS-CoV-2 into each city as a Poisson process with mean *I*. That is, we assume that each day prior to the quarantine, *I*[*t*] infected travelers arrive into a city where they begin infecting locals. We assume that on average, an infectious person infects *r* people each day, who in turn become infective (able to infect others) in one day. That is, on day *t* there are *I*[*t*] new infectious arrivals, as well as the *N*[*t* − 1] total infectious people from the day before, and the *rN*[*t* − 1] people they infected (Eq 1).

$$\begin{array}{c} N[t]=N[t-1](1+r)+I[t] \end{array}$$ (1)

This difference equation is easily solved. We find that the expected number of infections on day *t* is:

$$\begin{array}{c} \bar{N}\left[t\right]=I\left(1+1/r\right){\left(1+r\right)}^{t}-I/r \end{array}$$ (2)

Eq 1 gives the expected number of infections in a given city on day *t*. In our analysis, we allow both *I*, the drip rate, and *r*, the transmission rate, to vary by city.

While the disease is spreading, an infrastructure is being established to detect the disease, which results in a dynamic disease detection rate. As a simple but useful case, consider a logistic increase in the detection rate. We define the total probability of detecting an arbitrary infectious disease host on day *t* as:

$$\begin{array}{c} p\left[t\right]=\frac{p_{f}}{1+e^{-k(t-h)}} \end{array}$$ (3)

The detection rate increases from a small value (*p*(0) = *p*_*f*_/(1 + *e*^*kh*^)) at rate *k*, to a final detection capacity of *p*_*f*_ with half capacity reached on day *h*. The probability of detecting *c* cases of SARS-CoV-2 on day *t* is distributed as a Binomial distribution *B*(*N*[*t*], *p*(*t*), *c*(*t*)). The average (expected) number of infections detected on day *t* is then:

$$\begin{array}{c} \bar{c}\left[t\right]=N\left[t\right]p\left[t\right] \end{array}$$ (4)

When *r* is small (which covers all cases of interest), the time-derivative of the log of the expected number of confirmed cases (the expected case log-velocity) is:

$$\begin{array}{c} \frac{d\;\text{log}\;\bar{c}}{dt}\approx \frac{r}{1-e^{-rt}}+\frac{k}{1+e^{k(t-h)}} \end{array}$$ (5)

Note that the drip rate (*I*) and the overall detection rate (*p*_*f*_) have fallen out. Eq 5 is the basic equation that we use to fit the data. In the S1 File, we extend the drip model to include both varying drip rates and death and recovery.

### Data

COVID-19 data for Colombia were downloaded from the web:

1. case data from the *Instituto Nacional de Salud* [27]
2. testing protocols from the *Ministerio de salud* [28].

Weather and population data were downloaded from:

1. World Weather Online [29]
2. Population data from City Population dot de [30]

Transportation data were taken from the web:

1. Airport data from the *Aeronáutica Civil de Colombia* [31]
2. Public transportation data from *Moovit* [32]

### Data analysis

The complete data analysis procedure is as follows:

1. We began the analysis on March 10, 2020 four days after the confirmation of the first case in Bogotá D.C. We removed the first four days (which does not exclude any additional cases) to account for a large drop in testing that occurred during this time (Fig 1).
2. In order to estimate the expected count number from the daily count number, we smoothed the daily confirmed case counts with a seven day triangle function. We include days past the quarantine cutoff in the smoothing to avoid edge effects from the filter. The data were then trimmed to the interval March 10 through April 3, 2020 for Bogotá D.C. and April 7 for the remainder of the cities.
3. We computed the count log-velocity (see S1 File) by first taking the numerical derivative of the smoothed count data (via the Python routine numpy.gradient) and then by dividing the gradient by the smoothed count data.
4. We fit the count log-velocity to the drip model according to the routine presented in the S2 Fig in S1 File.

Since the data span was about 30 days, exponential growth will only be observable for cities with transmission rates significantly greater than 1/30 ∼ 0.033. Consequently, 0.05 was considered to be the minimum threshold on the transmission rate to observe exponential growth in a given city. We associate transmission rates above this threshold with airborne transmission and transmission rates below this threshold with tourism and direct transmission [33].

In order to deduce the cutoff date for the quarantine, we examined the case counts in Bogotá (S1 Fig in S1 File). Since we assume the transmission rate is constrained to be constant, the reduction in the transmission rate induced by the quarantine shows up in our model as a decrease in the detection rate (red vertical line). The vertical bar at April 3, 2020 denotes a combination of the impact of the quarantine on the spread of SARS-CoV-2 in Bogotá and a brief decline in the testing rate (Fig 1). Since Bogotá went into quarantine 4 days before the rest of the country, its pre-quarantine data ends April 7, 2020.

## Results

**Fig 2:**
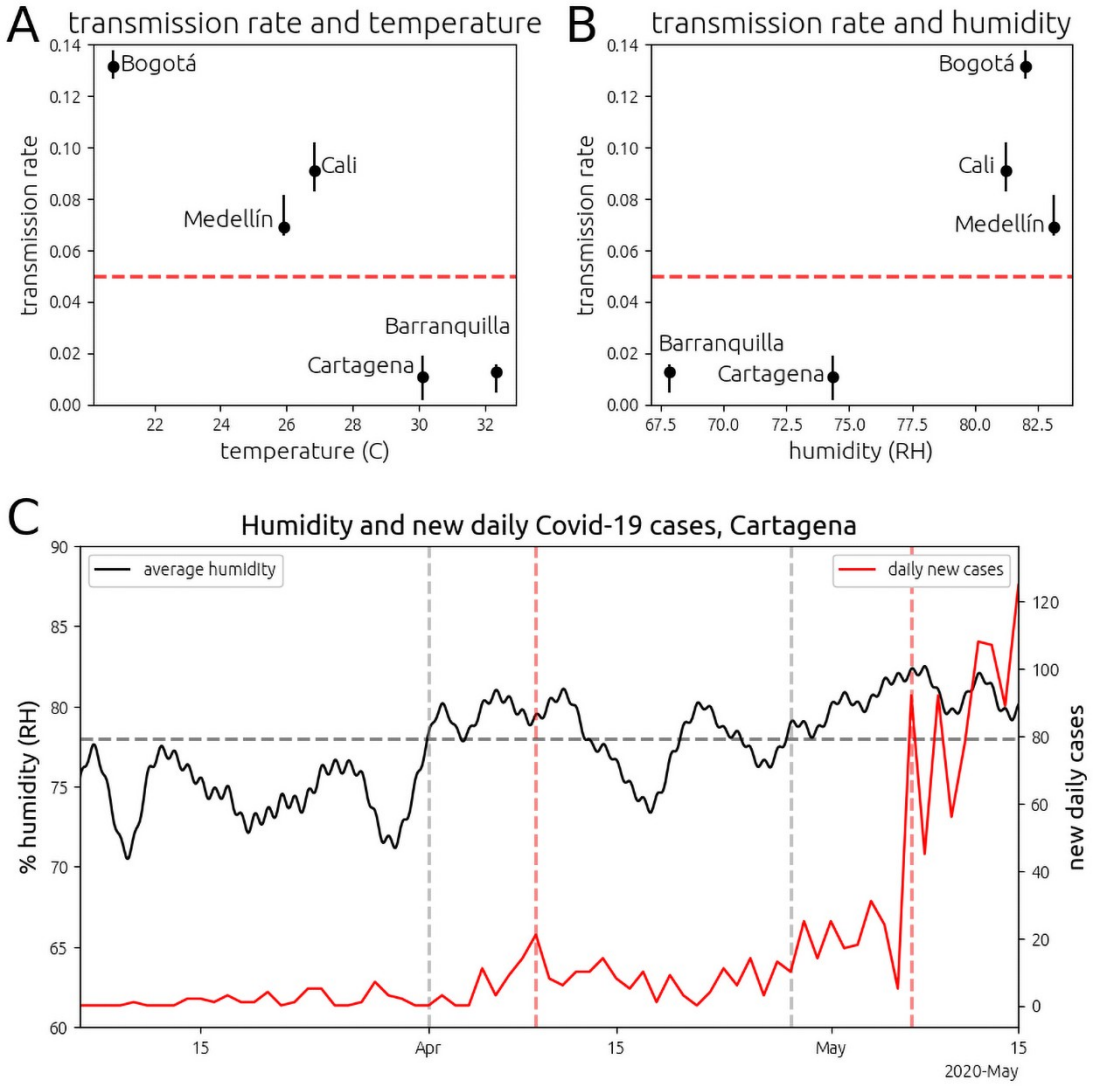
The transmission rate of SARS-CoV-2 with temperature and RH in the five largest cities of Colombia. (A,B) The dashed red line denotes a transmission rate of *r* = 0.05, which is our model’s threshold for observing clear exponential behavior and which we associate with airborne transmission. Note that these rates only apply to the pre-quarantine period. (C) Outbreak in Cartagena de Indias. The solid black line plots the average daily humidity in Cartagena (left axis). The solid red line plots the number of new cases of COVID-19 diagnosed in Cartagena (right axis). The horizontal dashed gray line plots a mean humidity level of 78%. The vertical dashed gray bar on the left denotes the first day with mean humidity at 78% since the first case of COVID-19 in Colombia. The first vertical dashed red line from the left, 8 days after the humidity increase, a spike in COVID-19 cases was registered. The second vertical dashed gray bar denotes the beginning of the second sustained period of humidity above 78% in Cartagena. A major outbreak began 9 days after the humidity rise.

**Fig 3:**
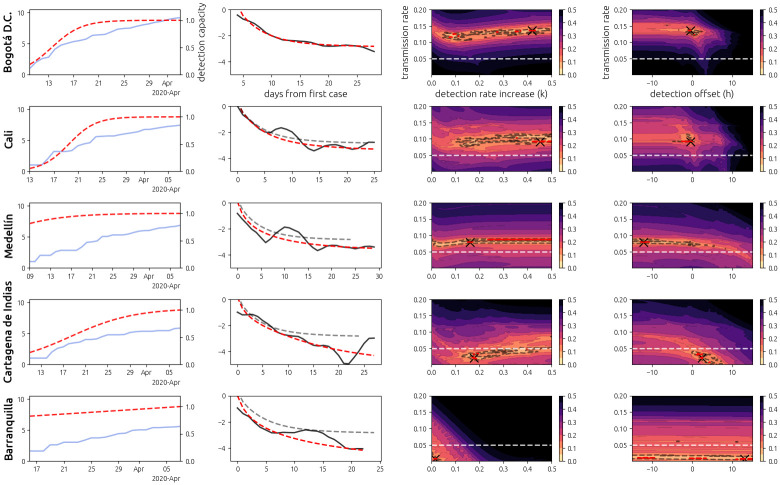
COVID-19 and model fit in the five largest cities of Colombia. Each row corresponds to a different city (left axis). The first column plots the cumulative number of confirmed cases of COVID-19 over the pre-quarantine period (blue) as well as the inferred detection capacity within the city (dashed red, right axis). The second column plots the log-velocity (see S1 File) of the data (black) and of the model fit (dashed red), with the model for Bogotá D.C. shown for reference (dashed gray). The third and fourth columns show cross-sections of the error function *e*(*r*, *k*, *h*) between the model under parameters (*r*, *k*, *h*) (transmission rate, detection rate increase rate, and detection rate half capacity date). The error bars are given in terms of percent increase from the global minimum. Global minima are denoted by a black cross. The dashed white line indicates the threshold *r* = 0.05 for observing clear exponential growth in our model. The red contours denote an increase from the global minimum of 5% and the gray contours denote an increase from the global minimum by 10%.

When we apply our model to the data, the data only shows clear exponential growth in large cities with Relative Humidity (RH) over 80% and mean maximum daily temperatures below 30 degrees Celsius (Bogotá, Cali, and Medellín—Fig 2). The RH in these cities is roughly the same (∼ 82%), and they have similar total populations and urban population densities (Table 1). The transmission rate declines among these cities with higher temperature. We also observe a fast rise in the detection rate (see S1 File) in the major cities (Fig 3).

We note that none of the smaller cities registered significant transmission rates in the pre-quarantine data at all temperatures and humidities. For example, Soacha and Bucaramanga compare to Bogotá and Medellín respectively. Soacha shares climate with Bogotá but has one tenth the total population (Soacha and Bogotá are neighboring cities and are connected by urban rail). Soacha has a higher urban population density than Bogotá. Bucaramanga has nearly one-fifth the population of Medellín (at similar temperature and humidity) and half the population density. Bogotá is more than 10 times larger than Soacha and Medellín more than twice as large as Bucaramanga. Moreover, Bogotá and Medellín are regional transportation hubs. Thus we conclude that the lack of transmission in Soacha and Bucaramanga is attributable to transportation factors as governed by total population and regional importance.

Although our analysis is focused upon pre-quarantine dynamics, there were two significant outbreaks post quarantine. The first was in Cartagena de Indias, a hot city on the Caribbean coast. While the weather in Colombia is nearly constant, there are minor temperature and humidity cycles associated with rainy and dry periods. The transmission rate in Cartagena correlates remarkably with sustained humidity above 78% (Fig 2). We conclude from this that the outbreaks in Cartagena were driven by changes in RH, since temperature was nearly constant, and Cartagena had been under quarantine, isolated from international travel and under tight local travel restrictions for 50 days prior to the outbreak. A second outbreak in a small town in the Amazon Rainforest, Leticia, on the banks of the Amazon river, resulted in confirmed infections in roughly 1 in 20 residents, the highest confirmed per-capita infection rate in Colombia. This outbreak is remarkable because Leticia, as a small town, does not have an urban city center or large buses or subways, and the mean daily RH is around 94%, the highest RH for a city in this study.

## Discussion

**Fig 4:**
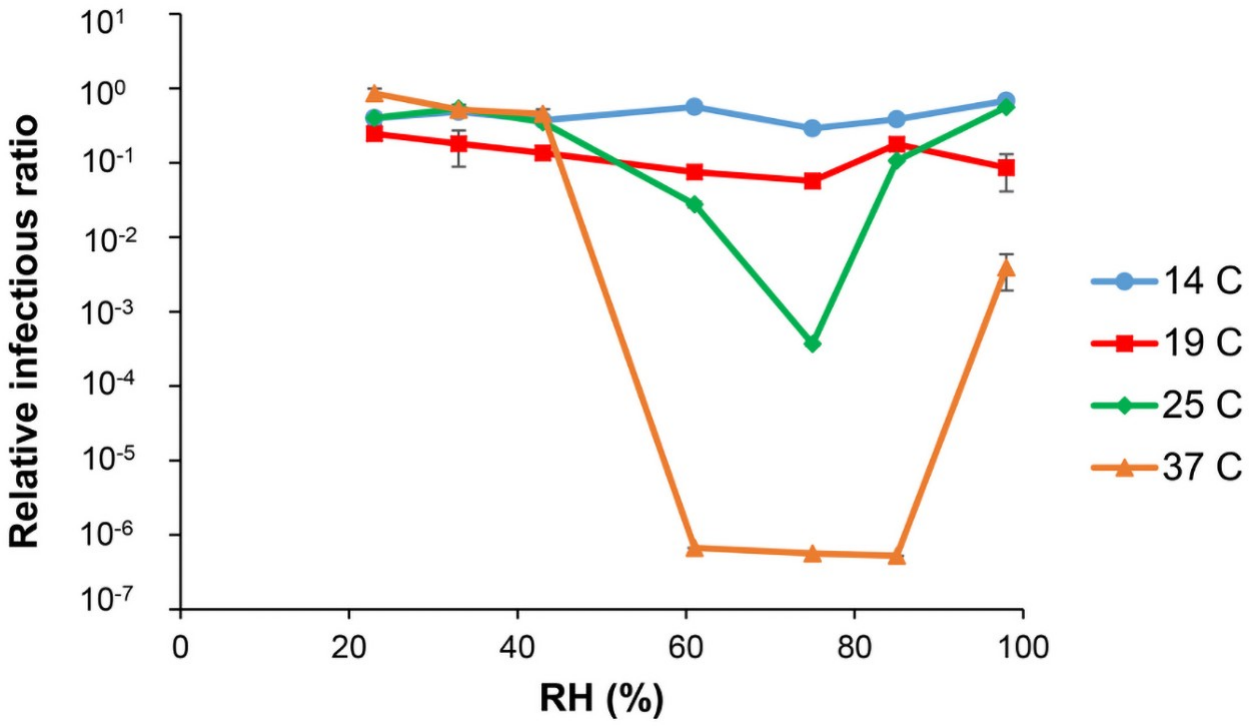
Phi6 infectivity with temperature and RH. Original data from Prussin et al [41] on the relationship between temperature, RH and Phi6 infectivity. Phi6 is an enveloped virus used as a model for influenza, coronavirus and other respiratory viruses. The error bars are shown but too small to be visible in the figure.

Comodulation of viral infectivity by temperature and by relative humidity has been experimentally demonstrated in a variety of enveloped viruses [34] such as SARS-CoV [35], influenza [16, 36–39] and SARS-CoV-2 [40] (for temperature only) and other enveloped viruses [41] (Fig 4). Three chief mechanisms have been proposed to explain the role of temperature and humidity upon enveloped viruses: *destabilization* of the virus within the droplet-matrix [36, 42], *evaporation and settling* of virus droplets [18, 42] and *reduced viral titer* produced by the host at higher temperatures [15].

Reduced viral titer could account for some of the reduced transmission observed at higher temperatures. For influenza, Halloran et al. [15] show a reduction in peak nasal titer for influenza by an order of magnitude for temperatures between 5 and 30 degrees Celsius in guinea pigs [15]. However, viral titer cannot explain the outbreak in Cartagena which appears to be driven entirely by high RH, since the air within the human respiratory system is saturated and unlikely to vary with small changes in ambient RH [43].

Although direct measurements at short timescales in conditions that mimic respiratory droplets are unavailable [42], temperature driven destabilization in solution apparently occurs on the timescale of minutes [36, 40, 41]. Droplet settling timescales vary from minutes to hours depending on droplet size [42]. Given the dense packing on public transportation in cities with attenuated transmission, we reason that the mechanism behind this attenuation must act on the timescale of seconds [15, 44] to be responsible for low infectivity. This rules out droplet settling and temperature driven viral destabilization as the cause of lower infection rates in these areas.

Airborne respiratory droplets evaporate down to half their size in about one second [37, 42]. This evaporation is thought to influence viral stability through salt and protein concentrations, pH gradients, and surface sheering [37, 42]. RH and temperature both influence droplet evaporation, with temperature influencing the rate and RH determining the final droplet size [15]. While the timescale of droplet shrinkage has been studied, the timescale of viral inactivation within the shrunken and toxic respiratory droplets is, to our knowledge, unknown [42]. Determining this timescale could have important implications for policy surrounding the COVID-19 pandemic, since fast destabilization at specific temperature and humidity intervals would have both prevention and therapeutic implications. Additional experiments are necessary to resolve the impact of temperature and humidity on the infectivity of SARS-CoV-2 within respiratory droplets.

In conclusion, we have discovered a qualitative dependence of the transmission of COVID-19 with climate. Furthermore, we have presented a mathematical model of disease spread that advances the understanding of disease transmission by addressing aspects of COVID-19 transmission that had not been anticipated in the literature. Additionally, we identified a plausible mechanism for the heat transmission barrier of COVID-19 transmission.

## Study limitations

Our study is limited by the simplicity of our model, the observational nature of the data, the relatively short duration of the observation, and the lack of information about the details of mobility and social interaction within the cities. Moreover, the climates observed only cover cool to warm temperatures and moderate to high humidities. Cold weather and dry climates were not observed in our study.

Despite these limitations, the results our of study are likely to be of general interest for several reasons. First, the transmission mechanisms of human respiratory virsuses are thought to be the same—particularly for enveloped viruses such as coronaviruses and influenza [14–19, 29–37]. Secondly, the impact of temperature and humidity upon these viruses is also thought to be the same (hence the seasonality of the common cold and influenza). Given the seasonality of illness-provoking enveloped viruses in humans, the main question that our article seeks to address is: does the evidence support qualitative agreement of the temperature and humidity dependence with other illness-provoking enveloped viruses (e.g. coronaviruses and influenza), or are the mutations that produced SARS-CoV-2 sufficient to have altered its response to climate?

Viewed in the this context, then, the problem is much simpler than it first seems. Our task is not to determine the entire temperature and humidity dependence of SARS-CoV-2 transmission, which would require careful measurements over a large range of temperature-humidity combinations. Instead, we observe the transmission of SARS-CoV-2 over a sampling of representative climates to determine if the transmission dependence agrees with existing models of climate dependence for other enveloped viruses. Colombia serves as the best country in the world for the analysis for the reasons mentioned in the Introduction: the near seasonless climate, the single, dominant port of entry, the conditions favorable to rapid spread (e.g. widespread use of public transport), and the swift and coordinated national response to the disease. Other countries near the equator, such as Brazil and Ecuador, had uncoordinated responses to the pandemic, with the national guidance in Brazil being particularly relaxed. In Ecuador, testing problems, including inadequate supply and lack of resources, render the data unreliable. Throughout Africa, poor record keeping and low case numbers prohibit reliable analysis.

In early March, there was almost no concern about COVID-19 in Colombia since the disease had almost no presence in all of South America. At that time, the major outbreaks were confined to China and Europe. Then, suddenly and preventively, a total quarantine was imposed in Colombia in response to WHO guidance. At that time there were still under 1,000 cases in Colombia (population 50 million). This sudden and total and preventative quarantine created a unique and opportune situation. During the time between the virus entry into Colombia and the quarantine, the country was functioning as normal since there was no concern about COVID-19 in the population. This created a steady state situation that is captured by our model.

The primary port of entry into Colombia is the international airport in Bogotá which feeds into a sprawling bus network that spans the entire country like a circulatory system, assuring that the disease was quickly spread throughout the country. Clearly, the rate at which the disease was carried into each city varied. A key aspect of our model is that it eliminates the rate at which the disease was delivered to each city (the drip rate). Applying our model to the pre-quarantine data, we were able to deduce how quickly the virus had spread within the different cities, removing the rate at which the virus was introduced into each city and removing the different rates at which testing was introduced into the cities.

The results of our analysis clearly suggest that there is broad, qualitative agreement of the transmission rates of SARS-CoV-2 with the Prussin model (Fig 4), which was developed to cover a broad range of infectious enveloped viruses such as Phi-6, coronavirus, influenza, etc. Again, this is not too surprising since, with some exceptions, there is general agreement that the mechanisms by which temperature and humidity regulate transmission arise from macroscopic viral properties, such as weight, size, surface PH, surface electrostatic potential, etc [42]. That is, the viral factors that regulate sensitivity to temperature and humidity are not likely to change dramatically among viruses of the same family that share a large fraction of identical genetic material and assembled structure [1, 2].

## Conclusion

Our observations are consistent with the Prussin data [41] on the infectivity of the Phi6 virus, which shows an exponential dependence upon *combined* temperature and RH values (Fig 4). This data suggests that temperature is the primary weather variable for transmission when the humidity is moderate. Above aound 20 degrees Celsius, infectivity is likely to increase exponentially with RH beginning around 75%. We therefore predict a decline in the spread of SARS-CoV-2 with temperature in regions with moderate humidity. We also predict an increased probability of outbreaks as the relative humidity approaches and surpasses 80% in warm and hot regions. Cities near large bodies of water, such as Beijing, where the humidity rises to around 80% in the summer, are at particular risk for outbreaks. Given the crowded public transportation systems and high urban population densities of cities with highly attenuated transmission, we hypothesize that the attenuation is caused by rapid viral inactivation within the respiratory droplet matrix as mediated by evaporation through temperature and RH, with direct temperature effects upon the virus contributing. Experimental confirmation of this hypothesis could have significant implications for policy surrounding the COVID-19 pandemic. For example, indoor climate control (or lack thereof) might be considered as a means of mitigating the spread of SARS-CoV-2. The same mechanisms of viral destabilization within evaporated respiratory droplets could be considered as a means of directly combating the virus. Establishing the timescale of viral destabilization within respiratory droplets, resolved on the shortest timescale possible, may then provide important information about the biology and transmission mechanisms of SARS-CoV-2.

## Supporting information

S1 File (PDF)
