# Supplementary material for: The transmission of SARS-CoV-2 is likely comodulated by temperature and by relative humidity
Source: PLoS One. 2021 Jul 29;16(7):e0255212. doi: 10.1371/journal.pone.0255212 (PMC8321224; doi:10.1371/journal.pone.0255212)
Supplement: S1 File — (PDF) [file pone.0255212.s001.pdf]

S1 File

As described in the main text, it is essential to distinguish the *transmission rate*, or the propensity of the disease to infect an exposed potential host, from both the total disease prevalence and the number of confirmed cases in a given population. Here we clarify the notions of detection rate, testing rate, and drip rate and quantify their impact upon the scaling of the confirmed case number and the apparent transmission rate.

The *detection rate* describes the total probability of detecting a case of SARS-CoV-2 in a given population. This rate undoubtedly increased over the period covered by our analysis since the testing rate increased by over two orders of magnitude during that time (Fig 1). In section *detection rate scaling*, we show that the detection rate introduces an additive factor into the exponent of the expected number of confirmed cases.

In section *drip rate scaling*, we show that the *drip rate*, or the rate at which infected travelers arrive into a city, introduces a multiplicative constant into the total number of infectious hosts in a given city which translates to a multiplicative constant in the expected number of confirmed cases.

In section *testing is driven by perception*, we demonstrate that the testing rate is not equivalent to the *detection rate*, since the testing rate is driven by human decisions

about going to the hospital, ordering tests, and testing protocols, whereas the *detection rate* describes the final probability of detecting a case of COVID-19. As an example, we show that panic can drive a wave of non-infected people to receive tests, driving up the testing rate, without changing the overall detection rate (subsection *panic in a disease free population*).

We make a distinction between the testing rate and the detection rate, because we use the observed exponential growth in the testing rate as motivation for including the detection rate in our data model, but do not introduce the testing data into our data analysis as a constraint, because our algorithm deduces the detection rate from the data without the need for constraints. Furthermore, our analysis shows that we should not expect the testing rate and the detection rate to have more than qualitative agreement.

In section *count log-velocity*, we show that the log-velocity, or the time-derivative of the log of the expected number of confirmed cases, is a useful measure of disease spread because it is independent of the drip rate and the overall detection rate. Since the log-velocity still depends on the detection rate dynamics, we include a logistic model for detection (section *drip model*).

detection rate scaling

Consider a population with a constant number N cases of COVID-19 with the total probability of detecting a case of COVID-19 written as $p[t]$. Since the probability of detecting c cases of COVID-19 is a binomial in the N cases, the expected number of *confirmed* cases on day t is:

$$\bar{c}[t] = Np[t] \tag{6}$$

When the detection probability is small, $p << 1$, the fluctuations in the observed case number are of order:

$$\sigma[t] = \sqrt{p[t](1 - p[t])/N} \approx \sqrt{p[t]/N} \tag{7}$$

Note that both the expected number of cases, $\bar{c}[t]$, and the fluctuation in the number of cases, $\sigma[t]$, are *increasing functions of the detection rate* $p[t]$. That is, both the measurement mean and the measurement variance depend on the detection rate. We assume that the function $p[t]$ can be well approximated by a logistic function. Hence,

$$p[t] = p_f/(1 + e^{-k(t-h)}) \tag{8}$$

Here the detection rate converges to a final rate p_f with rate k and reaches half the final rate on day h. In the early stage of the pandemic, while the detection capacity is a small fraction of its later capacity, we have $t < h$ and $\exp\{-k(t-h)\} >> 1$. Then, to first order:

$$p[t] \approx p_f e^{k(t-h)} \tag{9}$$

The log of the expected number of cases on day t is then:

$$\log \bar{c}[t] = kt + \log(Np_f e^{-kh}) \tag{10}$$

Eq 10 shows that the log of expected number of counts increases linearly with rate k. Thus, at the beginning of testing ramp-up, *constant infections with logistic growth in the detection rate is indistinguishable from exponential growth in the number of infections with a constant detection rate.* To see this, simply substitute $N = Ne^{rt}$ for the number of infections and substitute $p(t) = p_f$ for the detection rate. Then

$$\log \bar{c}[t] = rt + \log(Np_f) \quad (11)$$

Since p_f is initially unknown, the observed case dynamics will appear equivalent when the number of infections is constant and the detection rate grows logistically versus when the number of infections grows exponentially and the detection rate is constant.

drip rate scaling

In the previous section we illustrated that the local detection rate introduces an additive factor into the apparent transmission rate of the disease. In this subsection, we demonstrate that the drip rate introduces a multiplicative factor into the total number of infections in a given location under very general assumptions. *We only assume that in the early stage of the disease, the disease hosts are weakly-interacting.*

Under the weakly-interacting disease host assumption, the infected disease travelers who arrive in a given city create new pockets of infection. Since we assume that the disease hosts are weakly-interacting, a city with twice the drip rate will have twice the number of pockets in which the disease grows. As long as the weakly-interacting assumption is true, these pockets grow independently at the same rate, with no overlap. The total number of infections will then be the sum of infections over each of these disease pockets. In this way, the drip rate introduces a multiplicative factor into the expected number of infections regardless of the details of how the disease spreads.

We quantify this intuition in the following way. Let each person in a given location (i.e. city) be assigned a unique number $1, 2, \ldots N$ for N total people. Let $T[t]$ be the set of diseased travelers (τ) that arrive in this location on day t with $I[t] = |T[t]|$. Then $T[1] = \{\tau_{11}, \tau_{12}, \ldots, \tau_{1I[1]}\}$ and $T[2] = \{\tau_{21}, \tau_{22}, \ldots, t_{2I[2]}\}$. On average, each of the $I[t]$ travelers that arrive on day t infect $r << 1$ people per day. That is, on average every $1/r$ days, each traveler will infect one new person, and every subsequent $1/r$ days this new infectee will go on to infect another person, and so on. Consider the function $\lambda_{\tau_{jt'}}[t]$ that returns the unique identifier(s) of the person(s) infected by traveler $\tau_{jt'}$ on day t (i.e. the j-th traveler to arrive on day t'). Note we are only counting people who live in the location to be infectees and are not counting other travelers that the traveler infects. Then the function $\Lambda_{\tau_{jt'}}[t] = \bigcup_{s=t'}^{t} \lambda_{\tau_{jt'}}[s]$ denotes all people that the infected traveler $\tau_{jt'}$ has directly infected until day t.

Since we are considering the beginning stages of the disease and are neglecting re-infection, $\Lambda_{\tau_{jt'}}[t]$ must be disjoint for each of the travelers. That is, without reinfection, a person can only be infected by one traveler. Next, we consider the pool of people with whom each infectee interacts. Denote by $c_0[n]$ all the people whom person n, not a traveler, can potentially infect by direct interaction. These are people who are susceptible to the disease and who come into contact with the infectee (n). Now denote by $c_1[n]$ all of the people whom person n can potentially directly infect. That is, $c_1[n]$ is the union of $c_0[n]$ with each $c_0[n']$ for each of the people n' in $c_0[n]$. In this way, we can consider $c_2[n], c_3[n]$ and so on, each of these sets becoming larger and larger as the pool of potential infectees becomes larger and larger. We refer to each stage of disease transmission as a generation. That is, the people in the set $\Lambda_{\tau_{jt'}}$, each infected by traveler $\tau_{jt'}$ between day t' and t, are the first generation of infectees. Then the people that these infectees infect are the second generation, and so on.

For the early stages of the disease, we need not consider very many generations. The generation timescale is $1/r$ and so the time for the disease to progress to the k-th generation is $1/r^k$. For $r << 1$, this time quickly becomes very large.

Thus, our assumption of weakly interacting hosts amounts to the assumption that the number of generations is small and the social circles of the early generations of infectees from each traveler are approximately disjoint. Recall that the first generation

of people infected by traveler $\tau_{jt'}$ until day t is $\Lambda_{\tau_{jt'}}[t]$. Then the total set of people who are susceptible to a second generation infection is $c_0[t, \tau_{jt'}] = \bigcup_{n \in \Lambda_{\tau_{jt'}}[t]} c_0[n]$ and the total set of people who are susceptible to a third generation infection from traveler $\tau_{jt'}$ is $c_1[t, \tau_{jt'}] = \bigcup_{n \in \Lambda_{\tau_{jt'}}[t]} c_1[n]$ and so on.

Eventually, $|c_m[n]| = N$ and the extended social circle of an arbitrary person includes the entire city. But for m small, we assume that the social circles are approximately disjoint, meaning that the presence of more infectious travelers does not restrict the dynamics of the generations of infections that stem from each traveler. Let m be the maximum number of generations to which the disease spreads during the early stages. We assume that $|c_m[t, \tau_{jt'}] \bigcap c_m[t, \tau_{kt''}]| \approx 0$: the set of potential infectees descendant from each traveler, over the relevant number of generations, is approximately non-overlapping.

Under this assumption, the presence of more infectious travelers does not restrict the spread of the disease. Thus, at any given time, a city with twice the drip rate will have roughly twice the total number of infections as a city with the same transmission rate and half the drip rate. This is what we mean by the statement that the drip rate introduces a multiplicative constant into the total number of infections. We can express this symbolically as follows. Denote by $N_0[t]$ the average disease dynamics subsequent to the infection of one local resident ($N_0[0] = 1$) and denote by $\bar{n}[t]$ the number of infections that the average traveler spreads per day. Then the total number of infections on day t is roughly:

$$N[t] = \sum_{t'} \bar{n}[t - t'] N_0[t - t'] I[t'] \tag{12}$$

The expected value of this this quantity is easily seen to be:

$$\bar{N}[t] = I \sum_{t'} \bar{n}[t - t'] N_0[t - t'] = In[t - t_0] \tag{13}$$

Here we see that a multiplicative constant has been introduced. Note that we have not introduced a specific model for how the disease spreads. We have only assumed that the disease spreads independently.

From this we conclude that the total number of cases in a given city does not necessarily inform us about the propensity of the disease to spread in the environment of the city, especially for short timescales and in cities where the transmission rate is low. As we will show in the next section, *the dynamics of the count numbers* tell us more about the transmission rate than the absolute scale of the number of infections at any time.

count log-velocity

So far we have only considered (k), the rate at which the disease detection probability increases, and the drip rate (I), the rate at which the disease is introduced into a city. We have not yet considered the total probability of detecting a given case of COVID-19 at a given moment in time.

Consider two cities, city 1 and city 2, that are alike in all respects except for the overall detection rate p_f. Also assume that the disease transmits in both cities at the same rate ($r = r_1 = r_2$), the disease is introduced into the two cities at the same rate ($I = I_1 = I_2$), and the disease detection infrastructure grows at the same rate ($k = k_1 = k_2$).

It is not necessary to specify the model of the disease dynamics, as long as the assumption that the disease spreads independently from each infected person is true. There may be other parameters of the model that we do not specify here. Whatever

these may be, they are assumed to be equal for the two cities. Likewise, we do not specify a model of the detection rate increase in the two cities, we only assume that it is some function $f(t,k)$ such that

$$p[t] = p_{f,j} f(t,k) \tag{14}$$

There are no restrictions on f other than it depend upon some rate k parameter that governs the rate of change of $p[t]$. The function $f(t,k)$ could depend upon other parameters which we have not specified but which are assumed equal between the two cities.

The probability of detecting c cases of COVID-19 in either city is a binomial and thus the expected number of counts is (from Eq 15):

$$\bar{c}[t] = p_{f,j} I n[t-t_0] f(t,k) \tag{15}$$

Eq 15 explains why static count numbers convey little information about the transmission rate. Depending upon the total detection probability p_f and the drip rate, I, the total count number between two otherwise identical cities could vary (hypothetically) by an order of magnitude or more. Note that both $p_{f,j}$ and I fall out of the time derivative of the log of the expected number of cases :

$$\frac{d}{dt} \log \bar{c}[t] = \frac{\dot{n}}{n} + \frac{\dot{f}}{f} \tag{16}$$

testing is driven by perception

In Colombia, From February to April 2020, detection was conducted at hospitals on symptomatic patients. In order for a patient to be diagnosed as COVID-positive, three steps were required: (1) a symptomatic disease host must go to the hospital, (2) the attending physician must order a test and (3) the test must result positive.

These three steps are not independent. For example, a severely symptomatic host is more likely to go to the hospital and request attention than a weakly symptomatic host or an asymptomatic host. Likewise, a severely symptomatic host is more likely to have a doctor order a test and more likely to test positive for COVID-19 than a moderately symptomatic patient.

Consider the following simple model for testing. The expected number of *unique* tests conducted on day t, $N_T[t]$, as the product of the number of people in the city times the probability of being tested. Since being tested requires going to the hospital, we separate the probabilities by the product rule:

$$N_T[t] = N p(H \mid t) p(T \mid H, t) \tag{17}$$

This division is essential because it divides the dynamics into two distinct populations: the general population and the hospital population. The general population dynamics $p(H \mid t)$ are driven by self-perception of symptoms, whereas the hospital dynamics $p(T \mid H, t)$ are driven by physician perception of symptoms and hospital protocol.

Note that the number of tests conducted on a given day is not, in general, equal to the number of *unique* tests conducted on a given day. This is because most countries implement protocols that call for duplicate testing. For now, we will not consider duplicate testing since we are only interested in the basic formulation and scaling of the testing dynamics.

Here $p(H \mid t)$ denotes the probability of going to the hospital with COVID-related symptoms, which for now, are assumed to be the only requirements for a symptomatic

S1 Table. Symptoms grades for testing model.

symptoms grade	description	symptoms
0	asymptomatic	none
1	mild	common-cold like symptoms
2	severe	pneumonia-like symptoms, respiratory distress

person to be tested. We expand the probability of going to the hospital in terms of those who are infected (i) and those who are not infected ($\bar{i}$) with SARS-CoV-2: those who are not infected include those who have other infections, e.g. influenza.

$$p(H \mid t) = p(H \mid i, t)p(i \mid t) + p(H \mid \bar{i}, t)p(\bar{i} \mid t) \tag{18}$$

Note that many people who are not infected with SARS-CoV-2 will have symptoms consistent with COVID-19. These symptoms may result from (1) other respiratory viruses (2) other health problems and (3) mass psychogenic illness (Table in S1 Table). It is well documented in psychology literature that the suggestion of symptoms provokes symptoms in a large percentage of the population, particularly when group consensus is involved [1].

We further expand each of the hospital terms in Eq 18 in terms of the severity of the symptoms. We use a three-grade ranking of symptoms: asymptomatic (0), mildly symptomatic (1) and severely symptomatic (2) (see Table in S1 Table). We assume that only symptomatic patients show up at the hospital as COVID-19 testing candidates, and that the general population dynamics is driven by self-assessed symptoms (such as difficulty breathing and fatigue).

$$p(H \mid i, t) \approx h_1[t]p(1 \mid i, t) + h_2[t]p(2 \mid i, t) \tag{19}$$

The term $h_1[t] = p(H \mid 1, t)$ ($h_2[t] = p(H \mid 2, t)$) denotes the probability of going to the hospital given grade one or grade two symptoms. Here $p(H \mid 1, i, t) = p(H \mid 1, t)$. This is because the probability of going to the hospital cannot depend on the true disease status as the decision to go to the hospital is subjective: the individual is not certain of his/her actual disease status.

$$p(H \mid \bar{i}, t) \approx h_1[t]p(1 \mid \bar{i}) + h_2[t]p(2 \mid \bar{i}) \tag{20}$$

Combining and re-arranging terms:

$$p(H \mid t) = p_i[t]\{h_1[t]\Delta p_1[t] + h_2[t]\Delta p_2[t]\} + h_1[t]p(1 \mid \bar{i}) + h_2[t]p(2 \mid \bar{i}) \tag{21}$$

Here $\Delta p_1[t] = p(1 \mid i) - p(1 \mid \bar{i})$ is the difference in the probability of exhibiting grade one symptoms in the infected population (i) and the remainder of the general population ($\bar{i}$); $\Delta p_2[t]$ has an equivalent definition for grade two symptoms and $p_i[t]$ denotes the disease prevalence among the population.

Eq 21 shows that the general population dynamics is driven by two terms: an infected sub-population term and an uninfected sub-population term. Interestingly, the infected sub-population term is driven by the fraction of people infected in the total population, $p_i[t]$, and *the difference in symptom rate* between those who are infected i and those who are not infected $\bar{i}$. That is, if the rate of moderate and severe symptoms between those who are infected and those who are not infected were the same, the number of people who show up at the hospital would not change as the disease spread.

Whereas patient perception drives general population dynamics, physician perception drives hospital dynamics. We now expand the probability of being tested in terms of the severity of symptoms under the assumption that the physician does not

administer tests to asymptomatic patients and always administers tests to severely symptomatic patients:

$$p(T \mid H, t) = f_2[t] + f_1[t]T_1[t] \tag{22}$$

with $f_2[t] = p(2 \mid H, t)$, $f_1[t] = p(1|H, t)$ and $T_1[t] = p(T \mid 1, H, t)$. In other words, f_2 is the fraction of people at the hospital that the physician diagnoses as having grade two symptoms, f_1 is the fraction of people at the hospital that the physician diagnoses as having grade one symptoms and $T_1[t]$ is the probability of administering a test to a grade one symptom patient (as diagnosed by the physician).

Note that we are suppressing an important condition here for the sake of compact notation. Here $p(2 \mid H, t)$, for example, denotes the fraction of people at the hospital with grade two symptoms *according to the physician's perception.* We might denote this with a P for emphasis - e.g. $p(2 \mid H, t) = p(2 \mid H, t, P)$. We emphasize this distinction because it is tempting to apply Bayes' theorem to simplify these expressions, but doing so requires care and keeping track of the conditions that we have largely suppressed in this brief presentation.

We note two interesting cases.

panic in a disease free population

When there is no disease within the population, then $p_i[t] = 0$ for all times considered. In that case, the probability of going to the hospital among the general population is (from Eq 21):

$$p(H \mid t) = h_1[t]p(1 \mid \bar{i}) + h_2[t]p(2 \mid \bar{i}) \tag{23}$$

A rough upper bound order of magnitude estimate for the probabilities $p(1 \mid \bar{i}, t)$ and $p(2 \mid \bar{i}, t)$ is about 1% and 0.1% respectively. Consider that during a typical flu season, over 50 million Americans contract the flu. For a population of 350 million, that is one in seven. Given that peak flu season spans about three months, then order 0.1% of the population is infected per day (new infections) during the flu season. If we make the approximation that all infections are symptomatic and the symptoms last for five days, then about 0.5% of the population will have symptoms *only from influenza* consistent with COVID-19 on a given day of peak flu season. Further note that roughly 10% of the population goes to the hospital per year, or about 0.02% per day assuming a uniform distribution. Thus, if we have $h_1 \sim 0.01$ and $h_2 \sim 0.1$ we obtain the right order of magnitude for people going to the hospital.

The small order of these numbers is important because a jump in $h_1[t]$ or $h_2[t]$ or in the probability of perceiving symptoms ($p(1 \mid \bar{i}, t)$ and $p(2 \mid \bar{i}, t)$) can provoke a hospital rush. Recall that $h_1[t]$ is the probability of going to the hospital with moderate symptoms. As a crude but simplifying assumption, we make the approximation that in a panic, all people with moderate or severe symptoms will go to the hospital: h_1 and h_2 will both be of $O(1)$.

This, in the panic caused by the introduction of a new disease, the fraction of people in the general population who experience moderate symptoms will make a large jump. In numerous studies in the literature, mass psychogenic illness (MPI) has been shown to arise and give rise to real, measurable symptoms, even when there is no underlying disease. This phenomenon is more prevalent in women than men (particularly young women). There were several instances of young women spreading panic on social media in the USA when they did not have the disease (see, for example, [2]).

As a model of MPI, we introduce the following form for the spread of psychogenic symptoms:

$$p(1 \mid \bar{i}, t) = p_G e^{kt} + p_0 \quad (24)$$

Here $p_G \sim 1/N << p_0$ since the psychogenic symptoms usually begin with a single case. This model is consistent with numerous case studies that show a rapid spread of symptoms among peer-connected groups. In some cases, an entire factory or an entire military base is crippled overnight by the spread of spurious symptoms. Indeed, the only factor slowing down the spread of psychogenic symptoms is (1) the delay in the transmission of information among susceptibles and (2) the number of people in social groups, as proximity of a symptomatic appears to be a key factor in triggering psychogenic symptoms.

For simplicity, we are limiting psychogenic symptoms to moderate symptoms. We assume that for all times considered, including $t = 0$, $p(1 \mid \bar{i}, t) >> p(2 \mid \bar{i}, t)$, or that cases with moderate symptoms greatly outnumber cases with severe symptoms. Then to first order, the probability of going to the hospital is:

$$p(H \mid t) \approx p_G e^{kt} + p_0 \quad (25)$$

The total number of unique tests on day t is then:

$$N_T[t] \approx (e^{kt} + N_T[0])\{f_2[t] + f_1[t]T_1[t]\} \quad (26)$$

Recall that $T_1[t]$ is the probability of a test being administered to a person with moderate symptoms. We assume that under a panic, $T_1[t] \to 1$. In such a situation, the testing rate becomes

$$N_T[t] \approx (e^{kt} + N_T[0])F[t] \quad (27)$$

where $F[t]$ is the total fraction of the hospital patients that the attending physicians perceive as having symptoms (moderate or severe) consistent with COVID-19. In the early stages of a panic, $F[t]$ must be rapidly increasing, or perhaps constant if the medical staff is particularly stoic. In any event, $F[t]$ is non-decreasing. Thus, MPI can cause an exponential rise in the testing rate, even when there is no disease in the population. In such a situation, for any significant false positive rate, the number of positive test results will also exhibit exponential growth.

ignorance among a diseased population

Now consider the opposite case: a disease spreads rapidly throughout a population who fail to recognize the outbreak. Assume exponential growth of the disease prevalence in the population $p_i[t] = p_0 e^{rt}$ and assume that people with moderate symptoms do not go to the hospital $h_1[t] = 0$. Since there is no awareness of disease spread, $h_2[t] = h_2$ is a constant. Then the probability of going to the hospital is:

$$p(H \mid t) = h_2\{p_0 e^{rt} \Delta p_2[t] + p(2 \mid \bar{i})\} \quad (28)$$

We assume that $p_0 << p(2 \mid \bar{i})$ so that at $t = 0$ the vast majority of people with severe symptoms do not have the disease. Then we can consider two times, $t << T$ and $t >> T$ with $T = r^{-1} \ln(p(2 \mid \bar{i})/p_0)$. In the first case, $t << T$, the fraction of people going to the hospital is constant at $\sim h_2 p(2 \mid \bar{i})$. Then for times $t >> T$, the fraction of people going to the hospital grows exponentially $\sim h_2 p_0 \Delta p_2[t] e^{r(t-T)}$.

Since there is no change in disease awareness, the fraction of the people showing up at the hospital getting tested is a constant. That is $p(T \mid H, t) = F_0$. Then for times $t << T$ the fraction of the population tested is:

$$f[t] \approx h_2 p(2 \mid \bar{i}) F_0 \quad (29)$$

We have now shown that, at least for some period of time, the testing rate can be constant in a region of exponential disease growth and that, conversely, the testing rate (and potentially the number of disease diagnoses) can grow exponentially in a region where there is no disease presence. On this basis we assert that disease testing rates are driven by perception. Likewise note that the disease testing rate cannot be considered proportional to the disease prevalence rate $p_i[t]$.

The model in this section is simple. The main purpose of this section is to show that (1) testing represents a complex and dynamic social phenomenon and (2) testing cannot be considered to be proportional to disease prevalence and (3) testing is not proportional to the probability of detecting a disease host in a given population.

continuing the drip model

In (S4 fig), we plot simulations for the drip model presented above in Materials and methods. We estimate the standard deviation in the count number as:

$$\sigma[t] = \frac{\sqrt{I}}{r}[(1+a)(1+r)^t - a] \tag{30}$$

Here $a \sim 0.15$ is a numerical parameter. Eq 30 shows that the fluctuations in the count number vary exponentially with time. This is another reason to distrust absolute count numbers without dynamics: stochastic fluctuations alone can produce exponential variations in count number.

varying drip rate

The dynamics can be expressed purely in terms of the drip rate as follows:

$$N[t] = \sum_{j=0}^{t}(1+r)^j I[t-j] \tag{31}$$

From Eq 31 we see that (1) the early drip rate drives the dynamics since these have the largest weighting and (2) the number of infections is linear in the drip rate.

recovery and death

We now extend the drip model presented above to include recovery and death. We refer to this as "removal" of the infectious host or "resolution" of the infection. As of the time of writing, the distribution of recovery times for SARS-CoV-2 is not precisely known, but it varies between one week and one month and depends upon various factors such as host immune response. We model removal by subtracting a removal fraction from the number of infections on day t:

$$N[t] = N[t-1](1+r-c) + I[t] \tag{32}$$

Here c is the fraction of people in the pool of infectious hosts who recovered or died. This fraction c depends upon (1) time (2) the transmission rate r and (3) the distribution of removal times. We will clarify each of these points in what follows.

According to the drip model, the number of new infections added to the population on day t is:

$$n[t] = rN[t-1] + I[t] \tag{33}$$

The number of people that are removed from the pool of infectious hosts (i.e. the number of people who recover or die) on day t follows some unknown distribution of removal probabilities. Let $q[t]$ be the number of people removed on day t. Then:

$$q[t] = \sum_{t'=2}^{T_M} p[t']n[t-t'] \tag{34}$$

Here $p[0] = p[1] = 0$ since we only count infections that are infectious for at least one full day and T_M is the maximum infection duration. Note also that we are assuming that the travelers are recently infected. From Eq 32, we see that the fraction c on day t is defined as the ratio of the number of people removed on day t to the total number of infections on day $t-1$. That is

$$c(r,t) = \frac{q[t]}{N[t-1]} = \frac{\sum_{t'=2}^{T_M} p[t']n[t-t']}{N[t-1]} \tag{35}$$

From Eq 35 we observe the dependence of c upon the transmission rate r, the time t, and the distribution of removal times $p[t']$.

We estimate the initial order of c as follows. First, we approximate the distribution p in Eq 34 by a delta function. That is, we suppose that $p[T] = 1$ (i.e. all infections are resolved on day T). Then on day $t = T$, the first removals leave the infectious pool. The fraction of people that leave can be approximated as the ratio of the expected number of removals on day $t = T$ to the expected number of infectious hosts on day $N[T-1]$:

$$c[t=T] \approx \frac{\mathbf{E}n[t-T]}{\mathbf{E}N[t-1]} = \frac{r}{e^{rT}-1} \tag{36}$$

This equation is only valid at $t = T$. If $c << r$, then this estimate will remain valid for future times. Using estimates on the transmission characteristics of SARS-CoV-2 by one of our coauthors, we obtain an estimate for $T = 15.5$ days [3]. With this long infectious period, the impact of including recovery dynamics on the early spread within Colombia is not significant (S5 Fig).

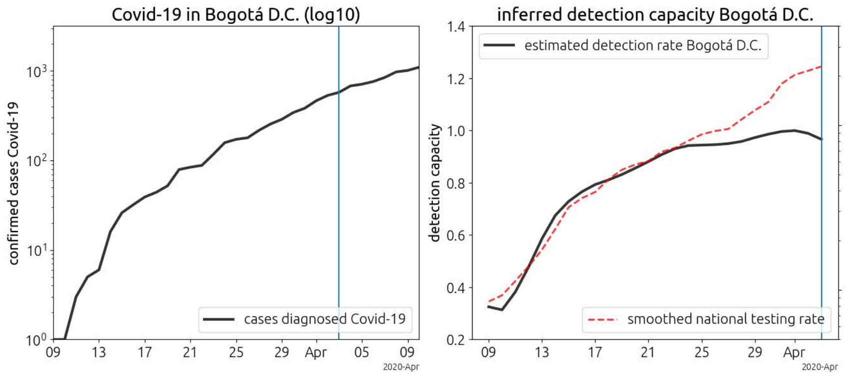

S1 Fig. S1 Fig. COVID-19 and testing in Bogotá D.C. In the right panel, the national testing rate (dashed red, log base 10) and inferred detection capacity for Bogotá D.C. only (black). Note we have plotted the smoothed national testing rate in dashed red; this plot is on a log10 scale and only serves to show qualitative agreement between the inferred detection rate and the daily testing rate. The national testing rate includes testing for all cities in Colombia.

```python
def fit_3param_model( count_log_vel, n_r=64, n_d=32,
                      r_max=0.2, h_max=15, k_max=.5) :
    n_days = len(count_log_vel)
    errors = np.zeros( (n_r, n_d, n_d) )
    r_vals = np.linspace( r_max/n_r, r_max, num=n_r )
    h_vals = np.linspace( -h_max, h_max, num=n_d )
    k_vals = np.linspace( 0., k_max, num=n_d )
    for u, r in enumerate(r_vals):
        infection_log_velocity = analytic(r, n_days, 1)
        detection_log_vel_plus_noise = count_log_vel - infection_log_velocity
        for v, k in enumerate(k_vals):
            for w, h in enumerate(h_vals):
                logistic_log_vel_k_h = logistic_log_vel( n_days, k=k, h=h )
                noise = detection_log_vel_plus_noise - logistic_log_vel_k_h
                errors[u,v,w] = np.sum( np.log(np.square( noise ) ) )

    u_star, v_star, w_star = np.unravel_index( np.argmin(errors), errors.shape )
    r_star, k_star, h_star = r_vals[u_star], k_vals[v_star], h_vals[w_star]
    return r_vals, k_vals, h_vals, r_star, k_star, h_star, errors
```

S2 Fig. Python code for conducting the fits. The first line grabs the number of days from the length of the input. The second line allocates memory for the error matrix based upon the number of parameters for the transmission rate (r) and the detection parameters (k,h). The next three lines compute the ranges of the parameters considered based upon input. The subsequent loops iterate over all model parameters in the following order: r, then k, then h. After each r, we compute the drip model for the infection log velocity via the function "analytic". From this we obtain the detection log velocity (or the empirical detection curve) by subtraction since the infection log velocity and the detection log velocity are additive in the count log velocity. In the detection loop, we compute the logistic curve for each (k,h) pair and subtract this from the detection log velocity to obtain the model noise. We compute the error as the log of the square of the noise. The log is required because the curve asymptotes to values near zero.

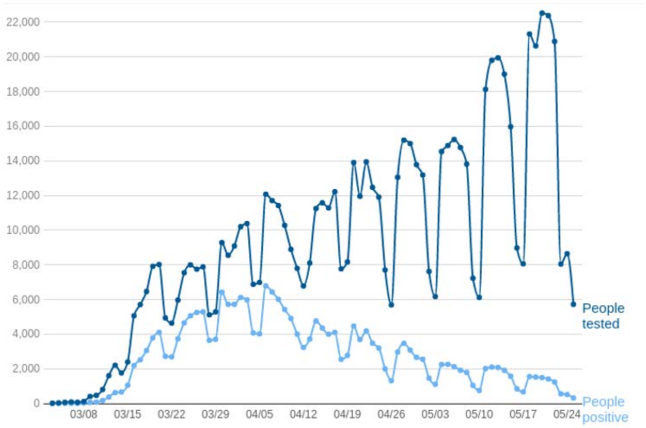

S3 Fig. Daily testing data from New York. Figure from the official website of the city of New York [4].

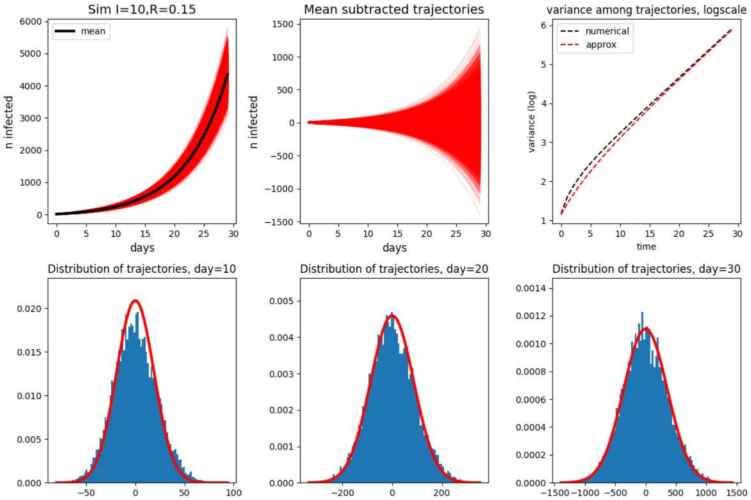

S4 Fig. Drip model simulation. Here $I = 10$ and $r = 0.15$. In the top row we plot (left panel) the trajectories (red) with the mean trajectory in black (computed from equation 2, (center panel) the mean-subtracted trajectories, (right panel) the standard deviation as computed numerically (black dashed line) and by equation 30 (red dashed line). In the bottom row, with plot the distribution of trajectories at day 10 (left), 20 (center) and 30 (right). The Gaussian fit in bold red in each of the plots in the bottom row is obtained by applying the mean from equation 2 and the standard deviation from Eq 30.

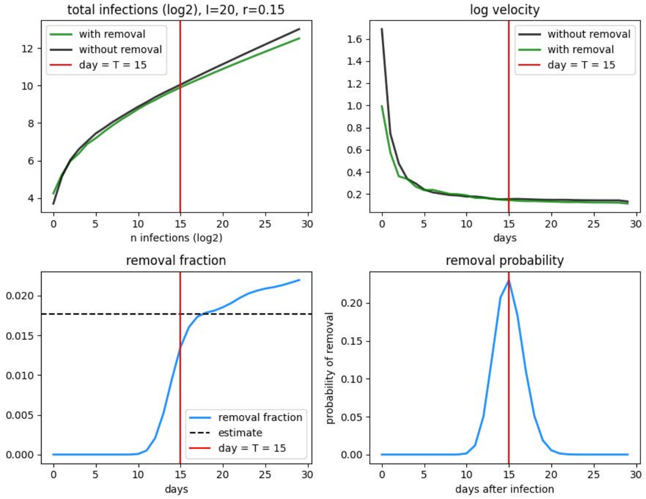

S5 Fig. S5 Fig. Drip model simulation with removal. We compare the dynamics for the drip model with and without removal. (upper left). The case counts for the two drip models with $r = 0.15$, $I = 20$ and $T = 15$. (upper right) The log-velocity of the two case counts considering removal (green) and without removal (black). (bottom left) The removal fraction (equation 36 computed numerically with the analytic estimate plotted in dashed black.) (bottom right) The removal probability used in the simulation: a discretized Gamma distribution with a mean of $T = 15$ days and a spread of 3 days.

References

1. Page LA, Keshishian C, Leonardi G, Murray V, Rubin GJ, Wessely S. Frequency and predictors of mass psychogenic illness. Epidemiology. 2010;21(5):744–747.

2. Zaveri M. Sheriff Told Teen to Take Down Posts About Coronavirus, Family's Lawsuit Says. The New York Times; April 21, 2020 [cited 2020 May 10]. Available from : https://www.nytimes.com/2020/04/21/us/marquette-county-sheriff-instagram-lawsuit.html

3. Bhanot G, DeLisi C. Predictions for Europe for the COVID-19 pandemic from a SIR model. medRxiv. 10.1101/2020.05.26.20114058 [Preprint] 2020 [cited June 1 2020]. Available from https://doi.org/10.1101/2020.05.26.20114058

4. COVID-19: Data; The Official Website of the City of New York [Internet] https://www1.nyc.gov/site/doh/covid/covid-19-data.page.
